# The Period Length of Fibroblast Circadian Gene Expression Varies Widely among Human Individuals

**DOI:** 10.1371/journal.pbio.0030338

**Published:** 2005-09-27

**Authors:** Steven A Brown, Fabienne Fleury-Olela, Emi Nagoshi, Conrad Hauser, Cristiana Juge, Christophe A Meier, Rachel Chicheportiche, Jean-Michel Dayer, Urs Albrecht, Ueli Schibler

**Affiliations:** **1** Department of Molecular Biology, University of Geneva, Geneva, Switzerland,; **2** Department of Biology—Howard Hughes Medical Institute, Brandeis University, Waltham, Massachusetts, United States of America,; **3** Division of Immunology and Allergy, University Hospital Geneva, Geneva, Switzerland,; **4** Endocrine Unit, Laboratory of Molecular Endocrinology, University Hospital Geneva, Geneva, Switzerland,; **5** Department of Medicine, Section of Biochemistry, Fribourg, Switzerland; Stanford UniversityUnited States of America

## Abstract

Mammalian circadian behavior is governed by a central clock in the suprachiasmatic nucleus of the brain hypothalamus, and its intrinsic period length is believed to affect the phase of daily activities. Measurement of this period length, normally accomplished by prolonged subject observation, is difficult and costly in humans. Because a circadian clock similar to that of the suprachiasmatic nucleus is present in most cell types, we were able to engineer a lentiviral circadian reporter that permits characterization of circadian rhythms in single skin biopsies. Using it, we have determined the period lengths of 19 human individuals. The average value from all subjects, 24.5 h, closely matches average values for human circadian physiology obtained in studies in which circadian period was assessed in the absence of the confounding effects of light input and sleep–wake cycle feedback. Nevertheless, the distribution of period lengths measured from biopsies from different individuals was wider than those reported for circadian physiology. A similar trend was observed when comparing wheel-running behavior with fibroblast period length in mouse strains containing circadian gene disruptions. In mice, inter-individual differences in fibroblast period length correlated with the period of running-wheel activity; in humans, fibroblasts from different individuals showed widely variant circadian periods. Given its robustness, the presented procedure should permit quantitative trait mapping of human period length.

## Abstract 2

A novel method permits characterization of circadian rhythms in humans and mice using single skin biopsies.

## Introduction

Circadian rhythms of physiology and behavior in mammals are dependent upon a central clock that resides in the suprachiasmatic nucleus (SCN) of the brain hypothalamus. This clock is synchronized to the outside world via light input from the retina, and it in turn entrains similar slave oscillators present in most cells of the body [1]. In constant darkness, the circadian clock will direct sleep–wake cycles and many other physiological processes according to its intrinsic period length, which may be longer or shorter than 24 h. Because the clock is reset by light each day, its intrinsic period length influences the relative phase of circadian physiology and activity patterns. Thus, in human beings there is a correlation between circadian period length and the entrained phase of physiological rhythms and sleep-wake timing [2,3]. Extremely early and late activity patterns are thought to be associated with advanced and delayed sleep phase syndromes, respectively. Both advanced and delayed sleep phase syndromes can have genetic causes, and polymorphisms in three circadian clock genes, *CK1*ɛ, *PER2,* and *PER3,* have been linked to or associated with cases of familial advanced or delayed sleep phase syndromes [4–6]. Polymorphisms in the latter gene have also been associated more generally with diurnal preference [7].

The characterization of human clocks and their genetic defects is rendered challenging by the difficulty and expense of measuring human circadian period, since prolonged subject observation under laboratory conditions is required. In mice, the period length of circadian behavior is determined by analysis of wheel-running behavior in constant darkness. Recently, however, it has been possible to complement mouse behavioral analyses by measuring the period length of circadian gene expression in vitro from transgenic animals in which the luciferase gene has been fused to a circadian promoter [8,9]. For these animals, circadian rhythms were analyzed in explants from different tissues simply by real-time measurement of light output. Using the same technology, high-amplitude circadian gene expression can also be measured in cultured mouse NIH 3T3 fibroblasts whose oscillators are synchronized through a short treatment with serum or dexamethasone, a glucocorticoid receptor agonist [10]. Moreover, single-cell recordings of cultured mouse and rat fibroblasts have demonstrated that the circadian oscillators of these cells are self-sustained and cell-autonomous [10,11], similar to those operative in SCN neurons [12,13]. The circadian rhythms of electrical firing frequencies of dissociated individual SCN neurons display considerable intercellular differences in period length (τ). However, the mean τ-values determined for neuron populations harvested from wild-type and tau mutant hamsters closely correlate with the ones measured for the locomotor activity of these animals [13]. Hence, the genetic makeup of the clockwork circuitry appears to influence cellular and behavioral oscillations in a similar fashion.

A method of measuring human circadian rhythms from tissue biopsies would greatly complement behavioral studies of circadian rhythms and the disorders affecting them, since genetic differences appear to manifest themselves in both central and peripheral oscillators [14,15]. In this paper, we employed a lentivirally delivered circadian reporter shielded by enhancer-blocking activities to achieve this result. The distribution of period lengths that we measured from 19 human subjects demonstrates that inter-individual genetic differences in circadian clock function can be measured in skin biopsies. In mice, clock function measured in this way correlated with the period of wheel-running behavior. In both organisms, the wide range of fibroblast circadian period lengths obtained suggests interesting differences between physiology controlled by the SCN and circadian gene expression directed by peripheral oscillators in vitro.

## Results

To measure circadian rhythms from a single biopsy, a circadian reporter must be introduced into the cells of a cultured tissue sample. However, human primary cultures do not easily permit transient transfection. Moreover, transiently transfected cells typically contain very high numbers of introduced reporter genes, an imbalance that can alter normal circadian rhythms by titrating circadian regulatory proteins [16]. Therefore, we turned to lentiviral delivery as a method of introducing a stably integrated construct in low copy number to primary fibroblast cultures [17]. We developed a lentivirus that contains a luciferase gene whose expression is governed by the promoter and 3′ untranslated regions of the mouse circadian gene *Bmal1*. To test this virus, it was used to infect immortalized 3T3 fibroblasts, and then circadian rhythms in these cells were synchronized by dexamethasone treatment [18]. BMAL1-luciferase expression was subsequently measured in the cell population by real-time recording of light output [9]. Cells infected with this virus gave high levels of expression, but very low circadian amplitude, so it was useless for circadian period measurements (data not shown).

Because lentiviruses integrate preferentially into the coding regions of active genes [19], we reasoned that the circadian behavior of the *Bmal1* promoter was hampered by interference from loci at which the virus integrated or, less likely, by viral sequences themselves. To shield the reporter gene from such influences, we introduced multimerized FII insulator sequences from the chick β-globin gene [20] upstream and downstream of the *Bmal1* reporter. These sequences have been previously shown to possess enhancer-blocking activity in vivo. When 3T3 fibroblasts were infected as above with these modified viruses, robust circadian oscillations were observed. As a parallel strategy, we introduced an “enhancer trap decoy,” consisting of the strong promoter of the human *Elongation Factor 1*α (*EF1*α) gene immediately followed by an SV40 transcription terminator, upstream of the *Bmal1* promoter. The resultant “decoy” construct also yielded excellent circadian oscillations of luciferase activity (Figure 1A and 1B). Because signal magnitude was consistently greater with it than with the insulated construct, it was used for the experiments described in this paper. To insure that the period lengths of the oscillations observed using this virus were not affected by the titer of virus used or by the degree of infection, 3T3 cells were infected with various amounts of virus, and circadian rhythms measured as above. The period length of the oscillations was identical in all cases, although signal amplitude varied over a wide range (Figure 1C).

**Figure 1:**
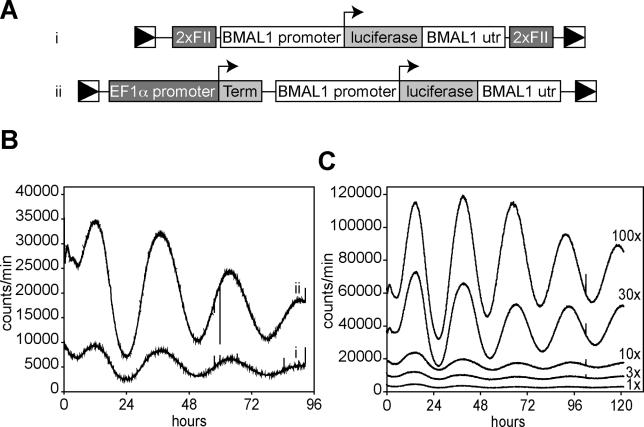
Circadian Bioluminescence Can Be Recorded in Fibroblasts Infected with a Lentiviral Luciferase Expression Vector (A) Circadian reporter constructs used in these studies. Each contains the mouse *Bmal1* promoter, the firefly luciferase coding region, and the *Bmal1* 3′UTR, flanked by the long terminal repeats (LTRs) of a lentiviral packaging vector. In (i), a dimerized chick β-globin FII element is inserted between each LTR and adjacent *Bmal1* sequences. In (ii), a DNA segment composed of the *EF1α* promoter and a SV40 terminator is inserted between the upstream LTR and *Bmal1* promoter, and the gfp coding region between the *Bmal1* UTR and the downstream viral LTR. (B) 3T3 cells were infected with the lentiviral vectors shown above, and equivalent infection levels were verified by real-time PCR to detect integrated viruses. Four days after infection, cells were shocked with dexamethasone to synchronize circadian rhythms, and luciferase output was measured by real-time luminometry. (C) 3T3 cells were infected with different concentrations of the lentiviral reporter vector ii (see [A]), and circadian rhythms were measured as in (B). 10× represents unconcentrated filtered viral supernatant, 1× represents a 10× dilution of this, and 100× was a 10× concentration by ultracentrifugation. The number of viral infection units/plate were approximately 10,000 (1×), 30,000 (3×), 100,000 (10×), 300,000 (30×), and 1,000,000 (100×).

Next, for two different individuals this virus was used to infect 50,000 activated human monocytes purified from a single blood donation, or 50,000 human fibroblasts amplified from a single 2-mm skin punch biopsy (see Materials and Methods for details). After 4 d, cellular rhythms were synchronized with dexamethasone, and luciferase output was measured. In both cell populations, circadian oscillations were observed; but with skin fibroblasts, we obtained much higher signals and greater amplitudes of circadian oscillation (Figure 2A and 2B); hence, more precise period lengths could be estimated. On rare occasions, it has also been possible to cultivate hair root keratinocytes that cling to the end of a plucked human hair. These keratinocytes can also be infected with lentivirus, and give period lengths identical to those from fibroblasts of the same subject (Figure 2C). However, because most plucked hairs do not contain keratinocytes without performing scalp biopsies, we decided to continue our analysis of human circadian rhythms using fibroblasts isolated from normal skin biopsies.

**Figure 2:**
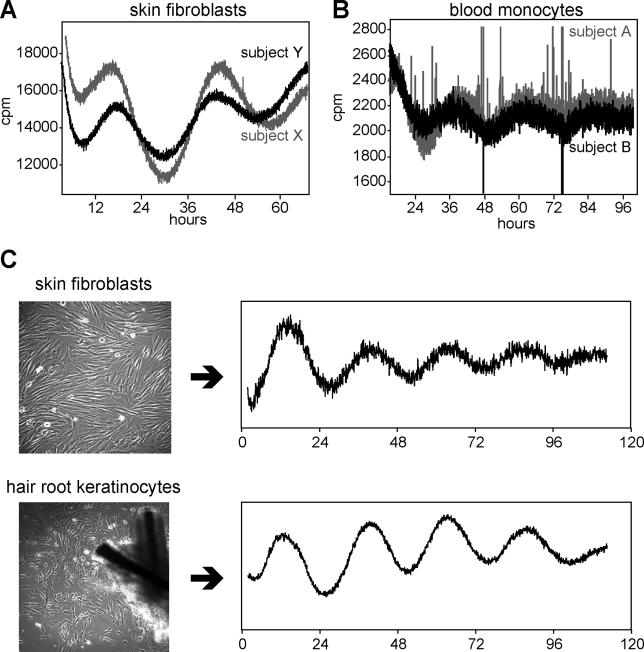
Circadian Bioluminescene Recordings from Primary Human Blood Cells, Fibroblasts, and Keratinocytes (A) For two different individuals, 50,000 non-immortalized adult primary human skin fibroblasts were infected with lentiviral reporter vectors. Four days after infection, cells were shocked with dexamethasone, and circadian rhythms were measured by real-time luminometry. (B) For two different individuals, 50,000 human blood monocytes were treated as in (A). (C) A skin biopsy was taken from one individual, and 50,000 fibroblasts were treated as in (A) and (B). In parallel, hairs were plucked until a hair was withdrawn that contained hair root keratinocytes clinging to the proximal end. These were cultivated and amplified to 50,000 cells, then infected and measured identically to fibroblasts.

To measure human circadian rhythms, two to five 2-mm diameter skin biopsies were taken from the abdomen or buttocks of 12 healthy normal individuals. Four additional human fibroblast populations were obtained from male foreskin, and three from other sources (see Materials and Methods for details.). From each of these 19 samples, 50,000 adult skin fibroblasts were infected with reporter virus, and circadian rhythms were measured as described previously. Two measurements on two infected populations from each biopsy were taken. Four sample curves are shown in Figure 3A, and the data are summarized in Figure 3B. An average period length of fibroblast circadian gene expression of 24.5 h was obtained, with a standard deviation of 45 min.

**Figure 3:**
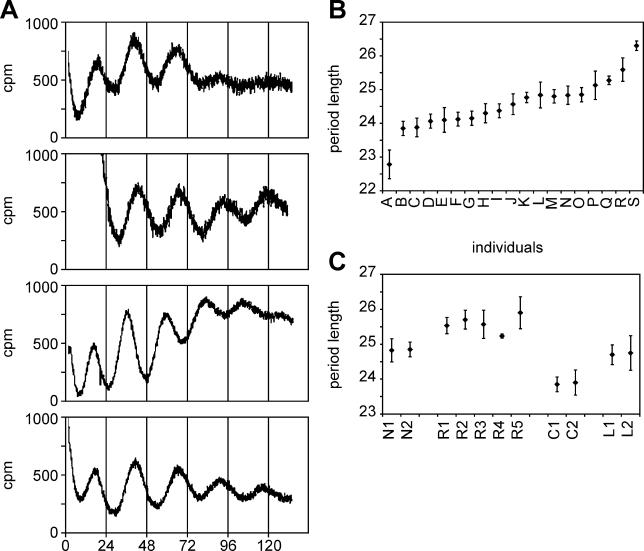
Circadian Bioluminescence Cycles in Fibroblasts from Different Human Individuals Biopsies were obtained from buttocks, foreskin, or abdomen of 19 individuals (see Materials and Methods for details). Fibroblasts were isolated from each biopsy, infected with lentiviral circadian reporter vectors as in Figure 2, and analyzed by real-time luminometry. Individuals are designated with the letters A–S. (A) Representative BMAL1-luciferase oscillations measured from biopsies of four different individuals. Individuals N, L, A, and P are shown. (B) Summary of the period lengths of BMAL-luciferase oscillations from all 19 individuals. Each value shows the average plus or minus the standard deviation from two different trials of two different infections of fibroblasts from two to five biopsies per subject. The probability by Student's *t*-test that the most different individuals (A and S) have the same period length is ≤0.00001; the probability that the second most different (B and R) are equal is ≤0.004. (C) For four subjects from whom two to five biopsies were taken, the average plus or minus the standard deviation of period length from two infections and four measurements of each skin biopsy is shown. The probability that the individuals that differ the most (C and R) do not differ in period length is ≤0.000002.

The period length of different cultures could in principle vary from biopsy to biopsy, or it could vary from individual to individual and remain constant among different biopsies of the same individual. Obviously, only in the latter case would the results be diagnostically useful. Hence, it was important to compare the range of data from different biopsies of the same individual with the range of data from different individuals. In multiple cases, inter-individual differences were significantly greater than the differences observed between cultures; four such examples and their statistical analyses are described in Figure 3C. Overall, the standard deviation among different trials using the same sample was 18 min; among samples derived from different infections of the same sample, the standard deviation was 25 min; and among the average values of different biopsies from the same person, the standard deviation was 6 mins. We conclude that this method can detect small differences in fibroblast circadian period length. What is particularly fascinating, however, is that the standard deviation among different individuals in our trial was 48 min. Thus, significant genetic differences in fibroblast clock function exist even in very small population samples.

To ensure that circadian genetic differences are indeed reflected in the rhythms of fibroblast gene expression that we measure, we applied this reporter system to measure circadian rhythms from tail biopsies of mice containing several known circadian mutations that shorten, lengthen, or abolish the period of circadian wheel-running behavior. Table 1 lists the mouse strains that we used and the published properties of their circadian clocks. We obtained adult dermal fibroblasts from tail biopsies of each of these nearly isogenic mice, and analyzed their circadian rhythms exactly as done in humans. Parallel to this analysis, circadian wheel running was measured for the same individuals (Figure 4). Mice with a period of wheel-running behavior shorter than wild-type *(Per1^brdm/brdm^)* yielded fibroblasts whose period of circadian *Bmal1* expression was also shorter. Similarly, fibroblasts from mice with a period of wheel running that was longer than wild-type *(Per1^brdm/brdm^*;*Cry2^−/−^,* and *Cry2^−/−^)* had correspondingly longer period lengths. Mice that were behaviorally arrhythmic *(Per2^brdm/brdm^, Per2^brdm/brdm^; Cry1^−/−^,* and *Per1^brdm/brdm^;Per2^brdm/brdm^)* produced arrhythmic fibroblasts. In most cases, however, the period of fibroblast gene expression was more extreme than that of behavior. For example, mice with behavioral periods shorter or longer than wild-type gave fibroblasts whose periods were even shorter or longer still. Similarly, mice containing the double disruption *Per2^brdm/brdm^;Cry2^−/−^,* which are behaviorally rhythmic and have a period of 24.4 h, yielded fibroblasts that typically show faint rhythmicity of 23–29 h for one cycle before becoming arrhythmic (Figures 4 and 5).

**Figure 4:**
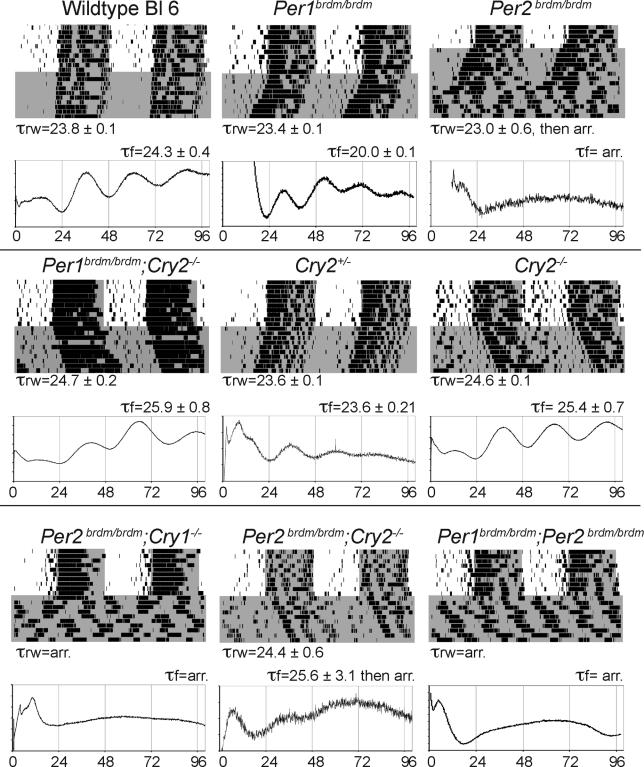
Comparison of the Period of Wheel-Running Behavior and of Fibroblast Bioluminescence among Mouse Strains Containing Different Circadian Gene Disruptions Mice of nine nearly isogenic genotypes were analyzed to obtain the period of running-wheel behavior (τrw) and the period of fibroblast luminescence (τf). For each genotype, a single running-wheel profile of the behavior of a single mouse kept first in normal light/dark conditions and then in constant darkness is shown. Periods of darkness are shaded on the graphs. Standard double-plotted actogram format is used, with consecutive rows representing consecutive days of activity, and *x*-axis showing time. Period lengths are presented below the graphs as average plus or minus the standard deviation for two mice, each measured twice. For each genotype, a single 100-h representative fibroblast recording is also shown. The *x*-axis shows time in hours; the *y-*axis shows arbitrary light units. Period lengths are presented above the graphs as averageplus or minus the standard deviation for two measurements of two biopsies of each mouse.

**Figure 5:**
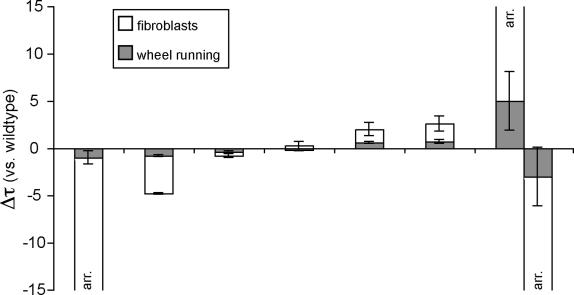
Genetic Differences in Circadian Period Length Measured from Fibroblasts Are Larger than Those Measured from Animal Behavior Data from Figure 4 depicted in stacked bar graph format. In each rhythmic strain measured, the period of wheel-running activity is shown in light grey, expressed in the difference in hours from the 24-h solar day. On top of this is shown the change in period of fibroblasts from the same animals, also measured in the difference in hours from the solar day. Genotypes depicted, from left to right, are *Per2^brdm/brdm^, Per1^brdm/brdm^,* wild-type, *Cry2^+/−^, Cry2^−/−^;Per1^brdm/brdm^, Cry2^−/−^, Per2^brdm/brdm^;Cry2^−/−^.* Because *Per2^brdm/brdm^;Cry2^−/−^* mice had unstable periods that ranged widely from individual to individual, this genotype is shown twice at the extreme right, with representative mice with periods both less than and greater than 24 h separated into two groups. Arrhythmic fibroblasts are designated “arr.”

**Table 1:**
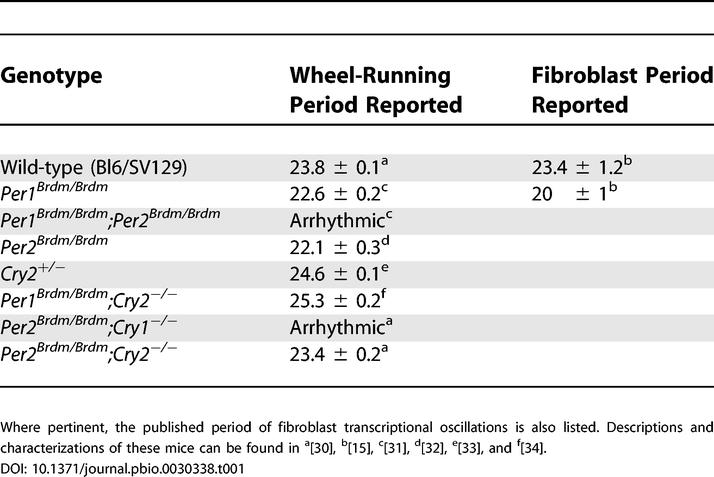
Mouse Strains for Whom the Circadian Period of Running-Wheel Behavior Was Compared with the Period of Fibroblast Bioluminescence

## Discussion

From the studies presented in this paper, we can conclude that molecular circadian rhythms can be measured in fibroblasts from skin biopsies and that the period of these rhythms is specific to an individual and can vary with genotype. Moreover, the variations observed among the 19 human individuals of this study suggest that the circadian clock is quite heterogeneous at a genetic level. The genetic origins of this variation will doubtless be a topic of future investigations, and our results suggest that fibroblasts could be an excellent system in which to investigate such differences by quantitative trait (QTL) mapping.

A major question posed by the research that we have presented is the relationship between fibroblast period length in vitro and the period length of human circadian physiology. Certainly, the two values are contingent upon the clocks of different tissues studied in different contexts (skin versus suprachiasmatic nucleus and in vitro versus in vivo). Although fibroblasts and SCN neurons possess clocks of very similar molecular mechanism [14], different mouse tissues from the same mouse can have periods varying by almost two hours when measured in tissue slices in vitro [8]. The average values that we obtained for the period of human circadian gene expression (24.5 h) correspond well with what has been published about rhythms of human circadian physiology (24.2–24.5 h) [21–24]. Values for period length in skin and fibroblasts of wild-type inbred mouse strains (23.5 ± 0.3 h) also corresponded nicely with behavioral and SCN period values obtained by us or published by others [8].

Nevertheless, our data suggest that it would be an error to assume that fibroblast period length is the same as physiologic period. Although on average the two corresponded well, significant differences were visible on an individual level. In mice, mutations at circadian loci that affected periodicity invariably had more extreme phenotypes upon fibroblast period than upon wheel-running period (Figures 4 and 5). Mice with behavioral periods 1 h shorter than wild-type gave fibroblasts whose period was 4 h shorter, and mice with periods 0.75 h longer had fibroblast periods 1.5 h longer. In most cases, though, longer or shorter periods of running-wheel behavior translate to longer or shorter fibroblast periods, respectively.

Our data hint at a similar disparity in humans. Specifically, the period lengths of human fibroblast gene expression showed inter-individual variation that was greater than what might have been expected from behavioral studies. Among our 19 human samples, a maximum difference of 4 h was seen, and six samples could be placed in categories that differ by more than 1.5 h. Altogether, a standard deviation of 0.8 h was observed. The period of circadian physiology measured by others in human beings showed standard deviations of 0.2–0.5 h under conditions of “forced desynchrony” during which circadian period was assessed in the absence of the confounding effects of light input and sleep-wake cycle feedback [21–24].

Although fibroblast clocks are not identical to SCN clocks, the fact that they use the same molecular components and that mutations at circadian loci affect biological timing in both tissues in the same qualitative fashion will doubtless render them quite useful in uncovering genetically-caused circadian differences among individuals and populations. Mammals other than humans can also show preferences of morningness and eveningness [25], and mouse wheel-running behavior has already been used as the basis for genome-wide quantitative trait analysis. In some human populations, chronotype questionnaires have suggested that morningness–eveningness tendencies can be widely distributed [26], and twin studies suggest that morningness and eveningness can be genetically determined [27]. Given its robustness, the presented procedure could be used in quantitative trait mapping of human period length and thus in the identification of genetic loci that participate in determining the period length and the phase of daily human rhythmicity. Ideally, studies with large numbers of human subjects should be performed with the least invasive cell harvesting techniques possible. Although the 2-mm cutaneous punch biopsies can be performed rapidly and heal completely within a few days, plucking hairs is obviously even less invasive. As shown in this paper, it was possible on occasion to harvest and cultivate primary keratinocytes from plucked hairs for the analysis of circadian gene expression. We hope that future efforts in optimizing this method will render it generally applicable.

## Materials and Methods

### Vector production

Figure 1A, construct (i): The *EF1α* promoter and *gfp* gene were removed from lentiviral backbone plasmid pWPI (http://www.tronolab.unige.ch, and replaced with a reporter cassette consisting of 1 kb of mouse *Bmal1* upstream region and 53 nucleotides of exon 1, fused in-frame to the luciferase coding region, and followed by 1 kb of *Bmal1* 3′UTR. Two chicken β-globin FII elements [20] were synthesized by PCR and inserted on either side of the reporter cassette. Construct (ii): The *Bmal1:luc* reporter cassette was inserted downstream of an *EF1α* promoter and SV40 terminator in pWPI. All viruses were produced, concentrated 10-fold by ultracentrifugation, and used for infection as described [28].

### Tissue isolation and culture

To establish our technique, five cylindrical 2-mm diameter cutaneous biopsies were taken from the ventral regions of five patients undergoing abdominoplasty operations. Subsequently, two biopsies were taken from the buttocks of each of seven recruited healthy adult subjects. Fibroblasts were isolated from biopsies by overnight digestion of tissue in DMEM/20% FCS/1 mg/ml collagenase type IA, and cultured in DMEM/20% FCS. Four foreskin fibroblast cultures were obtained by similar methods, and three other adult dermal fibroblast cultures were obtained from others (generous gift of S. Clarkson). Adult mouse fibroblasts from wild-type, *Per1^brdm/brdm^,* and *Per1^brdm/brdm^; Per2^brdm/brdm^* mice were isolated from 2-mm tail biopsies by the same method. Monocytes were isolated as described from fresh human blood from the Geneva University Hospital Blood Bank.[29]. Prior ethical consent for the use of all human tissues was given by the ethical committee of the Geneva University Hospital, informed consent was obtained from all human subjects, and animals were handled according to institutional guidelines.

### Synchronization and measurement of circadian rhythms

Four days or more after infection or cell passage, circadian rhythms were synchronized by dexamethasone [18]. Medium without phenol red was supplemented with 0.1 mM luciferin, and light output was measured in homemade light-tight atmosphere-controlled boxes for at least 4 d [9].

### Mouse running-wheel behavior

Mice of various genotypes were housed in cages with controlled lighting, each equipped with a running wheel (Mini Mitter, Bend, Oregon, United States) Running-wheel actograms and period determination were done with the Stanford Chronobiology kit (Stanford Software Systems, Stanford, California, United States).

### Statistical methods

For each luciferase measurement, the period of oscillation was calculated by fitting the curve to sine waves of known period using a macro program for Microsoft Excel written by SAB. The maxima and minima of each oscillation were identified, and the timing of these points was used to fit hypothetical sine curves with period and phase as free variables. The period of the sine wave with the best least-squares fit to the data was assumed to be the true period of oscillation. Because the period length of the first day after synchronization varied according to the conditions of synchronization, it was not included in these calculations; rather, period was determined by analyzing only days 2–5. To determine the period length of a particular biopsy, two independent viral infections were performed, and two synchronization/measurement cycles were done for each infection. To determine the period length of a particular individual, at least two separate biopsies were analyzed in this manner. Values are presented as mean plus or minus the standard deviation.

## Supporting Information

### Accession Numbers

The Genbank (http://www.ncbi.nlm.nih.gov/Genbank) accession numbers for the genes discussed in this paper are chick β-*globin* (V00409), human *CK1*ɛ (L37043), human *EF1*α (X03558), human *PER2* (AB002345), human *PER3* (AB047686), mouse *Bmal1* (Q9WTL8), mouse *Cry2* (AB003433), mouse *Per1* (AF022992), and mouse *Per2* (AF036893).

## Abbreviations

LTR: long terminal repeat
SCN: suprachiasmatic nucleus
